# Influence of bad oral habits upon the development of posterior crossbite in a preschool population

**DOI:** 10.1186/s12903-023-03572-0

**Published:** 2023-11-25

**Authors:** Antonio F. Galán-González, Antonia Domínguez-Reyes, M. Eugenia Cabrera-Domínguez

**Affiliations:** Department of Stomatology, Faculty of Dentistry, University of Seville, C/ Avicena s/n, Sevilla, 41009 Spain

**Keywords:** Crossbite, Oral habits, Malocclusion, Children

## Abstract

**Background:**

A study is made of posterior crossbite in deciduous dentition and its possible association to extrinsic factors (bad oral habits).

**Methods:**

A total of 1168 Spanish children between 3 and 6 years of age were included in the study. Exploration of the oral cavity was performed to assess the presence of crossbite (uni- or bilateral and/or functional), and a questionnaire was administered to the parents or caregivers to determine the presence of bad oral habits and their duration.

**Results:**

In occlusion, 19.7% of the cases (n = 230) presented uni- or bilateral posterior crossbite. On adopting centric relation confronting the midlines, crossbite persisted in 165 children, indicating that 65 cases were due to premature contacts (functional crossbite). The identified favoring factors were pacifier use, thumb sucking, oral breathing and tongue thrusting or immature swallowing.

**Discussion:**

Most studies in the literature report a relationship between posterior crossbite and bad oral habits. The proportion of posterior crossbites identified in our study (16.6%) is consistent with the data published by authors such as Kobayashi, Limeira or Paolantonio, among others, but differs from the results of Zhifei Zhou, Peres or Germa. In coincidence with most studies, we recorded a statistically significant association between posterior crossbite and bad oral habits.

**Conclusions:**

Bad oral habits favor the appearance of posterior crossbite, and the duration of the habit, its intensity (in the case of thumb sucking) and type (in the case of pacifier use) act as influencing factors. Functional study characterized the types of posterior crossbites and identified those attributable to premature contacts. This aspect has not been addressed by previous studies, and we consider the findings to be very interesting for analyzing and identifying the features of true crossbites.

## Introduction

Occlusion is currently considered to be a dynamic concept encompassing not only the teeth but also muscles, the temporomandibular joints and related tissues, and is intimately associated to the functions of the stomatognathic system (speech, breathing, swallowing and chewing), and to different neuromuscular, neuro-occlusal and postural factors of the individual. Thus, occlusion is conditioned by a number of factors, including the position of the tongue and teeth, the temporomandibular joints, the periodontium, the posterolateral muscle chain of the neck, the chewing muscles, pterygoid muscles, eruption disorders and ankylosis, supernumerary teeth, ectopic eruption, etc., as well as by other factors such as dental attrition, the type of diet, and bad oral habits [1].

In this regard, adequate maxillary growth will allow correct development of the dental arches and therefore contribute to good speech, swallowing, chewing and breathing functions. When adequate for the age of the individual, such functions result in good occlusion. Apart from optimum functioning of the stomatognathic system, the absence of factors interfering with normal occlusion (such as bad oral habits) is of crucial importance in these developmental stages [1, 2, 3].

Malocclusion is one of the most common disorders of the oral cavity [6], presenting a high incidence in children with mixed or permanent dentition [7–9].

Occlusion in deciduous dentition is defined by a series of characteristics and parameters that may be regarded as indicators of good occlusion in the future permanent dentition. In this respect, the upper teeth should extend beyond the lower teeth in the buccolingual direction, thereby causing occlusion of the lingual cusps of the upper molars in the anteroposterior sulcus separating the buccal cusps from the lingual cusps of the lower molars. The absence of such occlusion, with the lower molars extending beyond the upper molars, is known as crossbite [4, 5].

Crossbite typically is not attributable to a single cause but is conditioned by different factors ranging from simple environmental influences to complex inter-relationships between the genetic antecedents of the individual and his or her environmental context. Malocclusions in general are most likely the sum of both elements, i.e., they are derived from genetically conditioned occlusion that is worsened by functional forces, eating habits or habits that may affect development of the individual [10–12]. Likewise, genetically unfavorable profiles may be favorably modified by input from local factors during the development process. Thus, it may be affirmed that the causes underlying malocclusion in deciduous dentition involve both intrinsic factors conditioning growth and extrinsic or environmental factors [13–15]. Recent research points to a multifactorial origin of malocclusion, with variable influences of different both genetic and environmental factors. Attention therefore should focus on those occlusion-influencing factors that may be intervened upon, namely environmental factors that can exert a positive or negative effect on the orofacial structures and occlusion. In this regard, we considered it important to carry out a study to analyze the current prevalence of crossbite among preschool children in our setting, along with the frequency of bad oral habits, since it is in children of this age where problems of crossbite are more amenable to correction and may even be prevented through adequate programs designed to eradicate poor habits.

## Materials and methods

A cross-sectional study was carried out in the city of Seville (Spain), involving the random selection of three schools from each of its 6 healthcare districts – one corresponding to each socioeconomic level (low, medium and high). Out of a total of 1589 preschool children, only 1168 (647 girls and 521 boys) met all the inclusion criteria:

- Age between 3 and 6 years (both included).
- Enrolment in one of the selected schools.
- Delivery of the questionnaire addressed to the parents.
- Absence of systemic or genetic disorders (maxillary hypoplasia, mandibular hyperplasia, malformation syndromes, etc.) capable of influencing the results obtained.
- No present or past orthodontic treatment.
- Exclusive breastfeeding or mixed feeding with a bottle and anatomical teat, during a period of no more than 24 months.

A dentist carried out an intraoral exploration to determine the possible presence of uni- or bilateral crossbite in occlusion. Exploration was performed with the mandible in centric relation, aligning the midlines, to discard cases of crossbite due to premature contacts, and the types of crossbite were assessed. None of the publications in our review of the literature have performed this analysis, which we consider to be an important innovation of our study, since it allows us to exclude crossbites caused by premature contacts – many of which do not represent true crossbites and are amenable to simple treatment by trimming the points of contact.

The materials commonly employed in epidemiological studies on oral health were used in the explorations: intraoral mirrors, dental probes, tongue depressors, antiseptic solution, material containers, gloves, masks, paper towels and calibrators (calipers and millimetered rulers).

After completion of the exploration, the parents received a questionnaire evaluating a number of aspects, including feeding in the first months of life of the infant (breastfeeding or bottle) and any present or past oral habits, along with their frequency and duration. The parents completed the questionnaire following the instructions and under the observation of trained examiners. The different bad oral habits were explained by the examiners, and any doubts or queries of the participants were adequately resolved.

### Ethical approval and consent

The parents of the children were duly informed about the purpose of the study, and prior written informed consent was obtained in all cases. The study was approved by the Biomedical Research Ethics Committee of the Government of Andalusia (Ref. code: 0937-N-15).

### Statistical analysis

Qualitative variables were reported as frequencies and percentages, while quantitative variables were reported as the mean and standard deviation (SD). In the presence of normal data distribution, use was made of the chi-square test for qualitative variables and of analysis of variance (ANOVA) in the case of quantitative variables, in order to assess possible associations. The nonparametric Kruskal-Wallis test was used in the absence of normal data distribution. Statistical significance was considered for p < 0.05. The IBM SPSS Statistics para Windows, version 26 (IBM Corp., Armonk, N.Y., EE.UU.) was used throughout.

## Results

### Prevalence of crossbite

Crossbites were studied both in occlusion and following functional analysis. In occlusion, 80.3% of the cases (n = 938) presented no posterior crossbite, while some type of crossbite was identified in 19.7% of the cases (n = 230). The distribution of these 230 cases is shown in Table 1. Right-side crossbites were significantly more frequent in girls than in boys (p < 0.05).

**Table 1:** Relationship between crossbite in occlusion and gender distribution (statistically significant for right-side crossbite in girls. Chi-square test)

C.B. IN OCCLUSION	BOYS	GIRLS	TOTAL
**NO C.B.**	433 (83.3%)	505 (77.9%)	938 (80.3%)
**RIGHT C.B.**	35 (6.7%)	**69 (10.6%) (p = 0.0160)**	104 (8.9%)
**LEFT C.B.**	37 (7.1%)	53 (8.2%)	90 (7.7%)
**BILATERAL C.B.**	15 (2.9%)	21 (3.3%)	36 (3.1%)
**TOTAL**	520	648	1168

On the basis of the functional analysis, 65 of the aforementioned 230 cases (5.6% of the global sample) corresponded to premature contacts, while crossbite was seen to persist in 165 cases. The latter were distributed as follows: 54 children (4.6% of the total) presented right-side crossbite, 48 children (4.1% of the total) had left-side crossbite, and 63 children (5.4% of the total) presented bilateral crossbite. These conditions were slightly more common in girls, though statistical significance was not reached.

### Prevalence of bad oral habits

With regard to bad oral habits, pacifiers or dummies were used at some point in time by 958 of the children (82.0%); of these, 425 were boys (81.7% of the total boys) and 533 were girls (82.3% of the total girls).

In relation to the type of pacifier used presently or in the past, 333 children (34.8%) used an orthodontic pacifier while 625 children (65.2%) used a conventional (round) pacifier. The mean duration of use was 21.10 ± 11.89 months (Table 2). Considering maximum normal use to be 30 months, 154 children (13.2%) in our sample presented a duration of pacifier use we regard as harmful for correct development of the dental arches, with no significant gender differences.

**Table 2:** Relationship between the duration of pacifier use and gender distribution (nonsignificant)

DURATION OF USE	BOYS	GIRLS	TOTAL
**1–2 MONTHS**	153 (36.0%)	187 (35.2%)	340
**13–30 MONTHS**	214 (50.4%)	249 (46.8%)	463
**OVER 30 MONTHS**	58 (13.6%)	96 (18.0%)	154
**TOTAL**	425	532	957

**Fig. 1:**
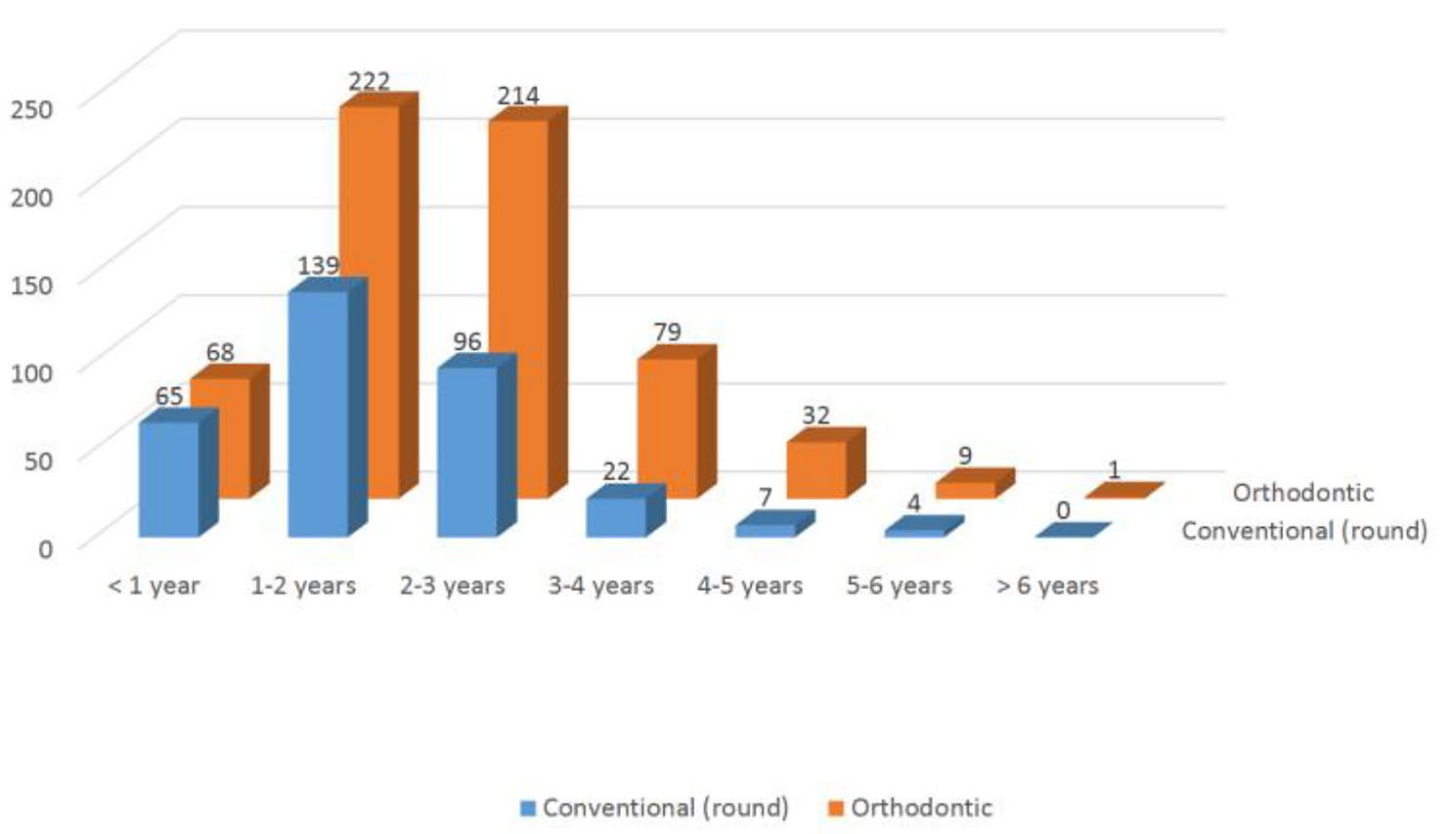
Duration of use and type of pacifier

In addition to identifying conventional pacifiers as being more widely used than orthodontic pacifiers, we explored the relationship between the duration of the habit and the type of pacifier, and found conventional pacifiers to be used for longer periods of time than orthodontic pacifiers (Fig. 1).

With regard to thumb sucking, of the total 1169 preschool children, 137 (11.7%) showed this habit at the time of the study or in the past. Thumb sucking was more frequent in girls (13.0% of the total girls) than in boys (10.2% of the total boys), through the difference was not statistically significant.

In relation to the duration of thumb sucking, 39 children (3.3% of the total sample) showed the habit for ≤ 12 months, 11 (0.9%) between 13 and 30 months, 29 (2.1%) between 30 months-4 years, and 57 (4.9%) for over four years. Some children even continued thumb sucking at the time of the exploration, thus evidencing the difficulty of permanently eradicating the habit.

With regard to the type of breathing (nasal, oral or mixed), the results of the parent questionnaire showed 650 children (55.7%) to sleep with the mouth closed (suggestive of nasal breathing) and 518 (44.3%) with the mouth open, while 479 (40.9%) snored. Of the children that slept with the mouth open, 371 (71.6%) snored, and 147 (28.4%) did not. Only 107 children (16.5%) that slept with the mouth closed were reported to snore.

Lastly, considering the environmental factors, we evaluated the prevalence of atypical or immature swallowing. On analyzing swallowing in the exploration, lingual interposition was observed in 309 children (26.5%).

### Association between the different oral habits and crossbite

A statistical analysis of the study variables was carried out to assess possible significant correlations between crossbite and the different oral habits. In this regard, we found pacifiers to be significantly associated to crossbite in occlusion (Table 3), and this applied to both pacifier use as such and to the type of pacifier and the duration of use, with a statistically significant relationship being found on considering right and left crossbites, but not bilateral crossbites. Crossbite was seen to be more common in children with the habit of pacifier use than in those who had never had the habit. However, on evaluating crossbite after the functional analysis, a statistically significant association was only observed with the duration of pacifier use.

**Table 3:** Relationship between the duration of pacifier use and crossbite in occlusion (statistically significant for right- and left-side crossbite; nonsignificant for bilateral crossbite. chi-square test)

C.B. IN OCCLUSION	NEVER PACIFIER	1–36 MONTHS	OVER 36 MONTHS
**NO C.B.**	185 (88.1%)	623 (77.6%)	40 (64.5%)
**RIGHT (p = 0,002)**	11 (5.2%)	76 (9.5%)	6 (9.7%)
**LEFT (p = 0,000)**	6 (2.9%)	62 (7.7%)	11 (17.7%)
**BILATERAL (no significativo)**	5 (2.4%)	25 (3.1%)	5 (8.1%)
**TOTAL**	210	803	62

With regard to thumb sucking, a statistically significant association was found with crossbite in occlusion, though only on the right side of the mouth.

**Fig. 2:**
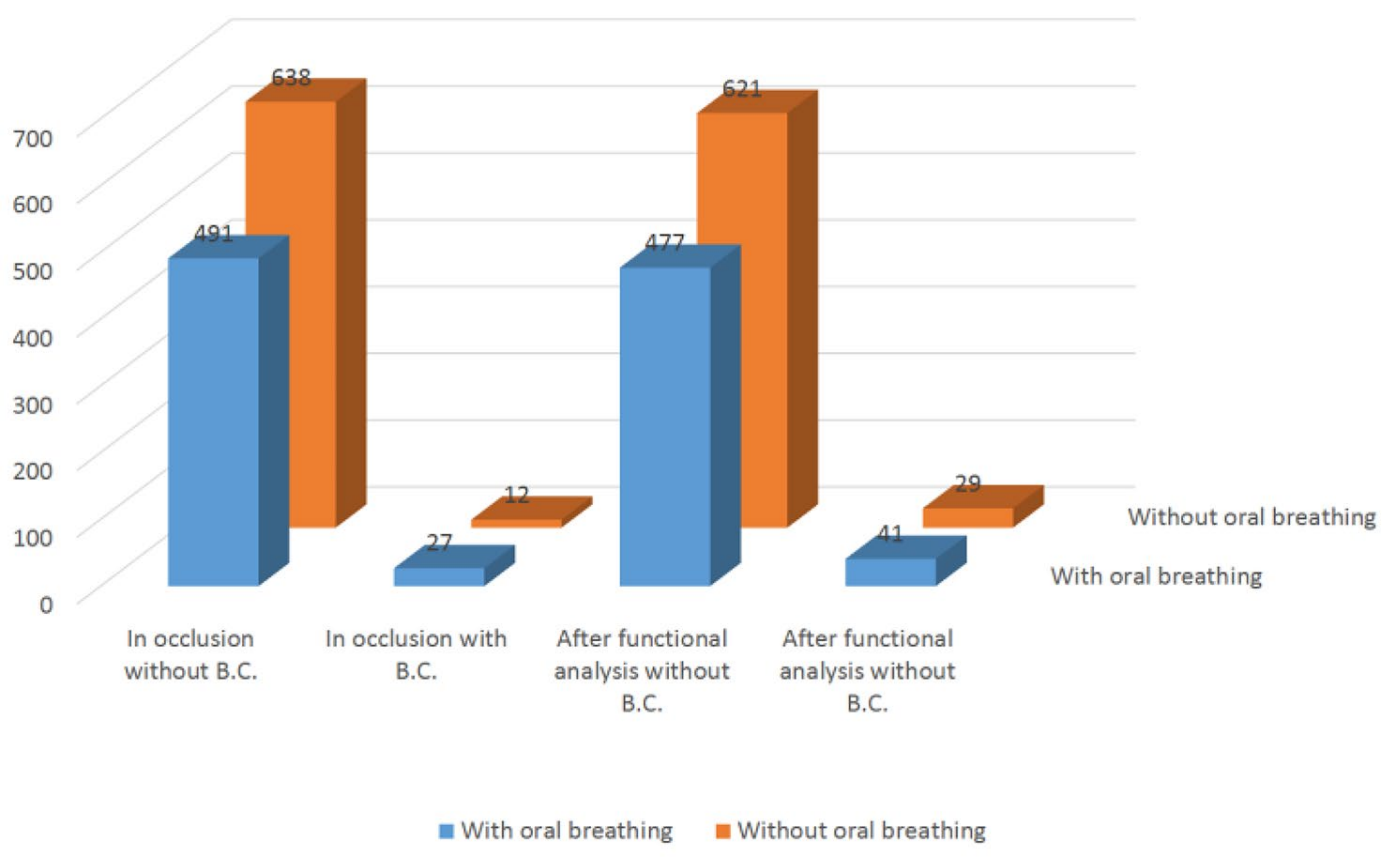
Bilateral crossbite and oral breathing

Oral breathing showed a statistically significant association to unilateral left-side and bilateral crossbite in occlusion. However, following the functional analysis, significance was only observed in the case of bilateral crossbite (Fig. 2).

On examining immature swallowing, we recorded a statistically significant increase in the presence of unilateral (both right and left side) crossbite in occlusion, but not in the presence of bilateral crossbite. Nevertheless, following the functional analysis, statistical significance was observed in relation to both unilateral and bilateral crossbite.

## Discussion

There are not many studies on the state of occlusion in deciduous dentition, the frequency of crossbite and/or its association to so-called bad oral habits. In contrast, many diverse studies have analyzed the types of occlusion and/or malocclusion in permanent dentition. Although some authors have reported no significant relationship between oral habits and posterior crossbite [10], the majority of published articles do describe such a relationship [11, 12, 13, 24, 26].

None of the consulted publications in the literature have adopted our analysis to determine the type of crossbite aligning both upper and lower midlines; we therefore have had to compare the results obtained in reference to crossbite in occlusion.

In relation to unilateral crossbite, our recorded percentage of 16.6% is consistent with the figure reported by Kobayashi (16.6%) [17], and not far from the values obtained by Ovsenik (14.0%) [15], Limeira (15%) [16] or Paolantonio (15%) [12]. Lower percentages have been reported by Bandeira (10.4%) [14] and Zhifei Zhou (7.56%) [18] or Wagner (3.4%) [19], while values higher than our own have been published by Peres (18.2%) [20] and Germa (20%) [22].

The slight predominance of right- over left-side crossbite in our sample (8.86% versus 7.71%, respectively) has also been described by Gonzalez-Cuesta [21] in the preschool population of Barcelona (Spain), though with figures of 12.44% (right side) and 7.37% (left side).

With regard to bilateral crossbite, we recorded a prevalence of 3.1%, while Ovsenik [15] documented 1.2% in 5-year-old Slovenian children.

In sum, overall posterior crossbite (uni- or bilateral), with a prevalence of 19.7% in our study, has been one of the parameters most consistent with the data found in the literature, with prevalences of 20% according to Ovsenik [15], 23.7% in the study published by Gonzalez-Cuesta [21], and 18.2% according to Peres [20]. Nevertheless, lower figures have been reported by Zhifei Zhou (7.56%) [18] and Bandeira (10.4%) [14].

With regard to bad oral habits, Scudine et al. [23] specifically addressed the relationship between malocclusions of this kind and pacifier use. In this respect, the prevalence of posterior crossbite in the children that had used a pacifier for over 36 months was significantly greater than in the children that had never used a pacifier (35.3% versus only 12.0%, respectively). It is also interesting to note that 22.3% of the preschool children that used a pacifier for less than 36 months also presented such crossbite. In this respect, we found that the longer the use of a pacifier, the greater the incidence of crossbite – thus suggesting a direct relationship between this habit and posterior crossbite. Melink [24] observed an increase in the number of crossbites from 18 months of pacifier use; Warren [25] reported that the longer the duration of the habit, the greater the frequency of malocclusion; and Peres [20] documented posterior crossbite in 16.3% of the children that had used a pacifier for 1–4 years, versus in only 1.9% of the children that had not used a pacifier or had done so for less than a year. Germa [22], Lopes Freire [26], Paolantonio [12], Ovsenik [15], Tomita [27] and Dimberg [28] likewise observed a relationship between posterior crossbite and pacifier use.

In concordance with the classical study of Swinehart [29], the occlusal alterations described by most authors as a consequence of thumb sucking appear to be attributable to both the passive force exerted by the thumb or finger between the arches and the anomalous contraction of the cheeks against the buccal surfaces of the teeth, as well as the muscle pressure exerted by the digit against the palate. These alterations in turn are conditioned by parameters such as the intensity and frequency of the habit, the thumb or fingers involved, the position of the digit within the mouth, and the position of the mandible during thumb or finger sucking [30, 31].

In relation to the transverse plane, and in coincidence with our own findings, different authors [30–32] have recorded an increased frequency of posterior crossbite in digit sucking children – this association being statistically significant in our study sample.

With regard to oral breathing, the accepted definition is that proposed by Ricketts [33], who described “respiratory obstruction syndrome” as being characterized by maxillary compression and posterior crossbite, protrusion of the upper arch, a low position of the tongue and lingual interposition. Our findings are very consistent with the observations of this author, with the recording of a greater frequency of crossbite (bilateral more than bilateral) in oral breathers – this appearing to be coherent with the mentioned maxillary compression, which has also been reported by other authors [15, 16, 20, 30].

Lastly, with respect to immature swallowing, the maxillary compressions described by most authors in patients of this kind [34, 35, 36] are usually clinically reflected in the form of posterior crossbite. Crossbite in our sample was seen to be increased not only on assessing the situation in occlusion but also and significantly on considering the functional analysis.

Our study is not without limitations. On considering the bad oral habits, we did not exclude those children with more than one habit; the influence of a given oral habit upon crossbite therefore may have been altered by this fact. On the other hand, the lack of published studies on crossbite with functional analysis prevented us from comparing our results with those of other investigators. Likewise, and although the parents received adequate information about bad oral habits and how to detect them, there may have been some subjectiveness in reporting of the required study information.

## Conclusions

The presence of bad oral habits produces early occlusal alterations in children. Patients with oral breathing and immature swallowing tend to have dental occlusion disorders.

The duration of the habit is an important conditioning factor of crossbite, especially in the pacifier habit, in the same way as the intensity of the habit in the case of thumb sucking, or the type of teat of the pacifier used.

The early detection and treatment of crossbite is crucial in order to avoid malformations of greater importance. It is advisable to eliminate bad oral habits at an early age in order to avoid severe occlusal repercussions.

## Data Availability

The entire dataset on which the conclusions of the study are based is presented in the principal manuscript. The related information is available upon request. You can request it by email: adominre@us.es.
